# An Overview of Vaccine Adjuvants: Current Evidence and Future Perspectives

**DOI:** 10.3390/vaccines10050819

**Published:** 2022-05-22

**Authors:** Alessio Facciolà, Giuseppa Visalli, Antonio Laganà, Angela Di Pietro

**Affiliations:** 1 Department of Biomedical and Dental Sciences and Morphofunctional Imaging, University of Messina, 98125 Messina, Italy; gvisalli@unime.it (G.V.); antonio.lagana1@studenti.unime.it (A.L.); adipietr@unime.it (A.D.P.); 2 Multi-Specialist Clinical Institute for Orthopaedic Trauma Care (COT), 98124 Messina, Italy

**Keywords:** vaccination, adjuvants, modern technologies, future perspectives

## Abstract

Vaccinations are one of the most important preventive tools against infectious diseases. Over time, many different types of vaccines have been developed concerning the antigen component. Adjuvants are essential elements that increase the efficacy of vaccination practises through many different actions, especially acting as carriers, depots, and stimulators of immune responses. For many years, few adjuvants have been included in vaccines, with aluminium salts being the most commonly used adjuvant. However, recent research has focused its attention on many different new compounds with effective adjuvant properties and improved safety. Modern technologies such as nanotechnologies and molecular biology have forcefully entered the production processes of both antigen and adjuvant components, thereby improving vaccine efficacy. Microparticles, emulsions, and immune stimulators are currently in the spotlight for their huge potential in vaccine production. Although studies have reported some potential side effects of vaccine adjuvants such as the recently recognised ASIA syndrome, the huge worth of vaccines remains unquestionable. Indeed, the recent COVID-19 pandemic has highlighted the importance of vaccines, especially in regard to managing future potential pandemics. In this field, research into adjuvants could play a leading role in the production of increasingly effective vaccines.

## 1. Introduction

Vaccinations are surely one of the most striking health achievements of human history. In just over two centuries, vaccines have allowed us to reach extraordinary goals such as the total eradication of smallpox, the disappearance of poliomyelitis in much of the world, and a strong decline in the mortality and morbidity of many infectious diseases in several countries [1]. In many parts of the world, vaccination policies are a public health cornerstone and great attention is paid to guarantee safe and effective vaccines to population [2,3,4]. The efficacy of a vaccine depends not only on the antigen components, but also on adjuvants that are often used in order to stimulate the immune system in a more effective way. Adjuvants are defined as constituents added to vaccines in order to improve immune responses towards an antigen. In addition, adjuvants have several benefits, such as the reduction in the antigen amount per vaccine dose and the number of vaccination sessions, and in certain cases, they increase the stability of the antigen component, extending its half-life and indirectly improving its immunogenic power [5]. Many different types of adjuvants are now available to use in vaccine manufacturing (Table 1).

**Figure 1:**
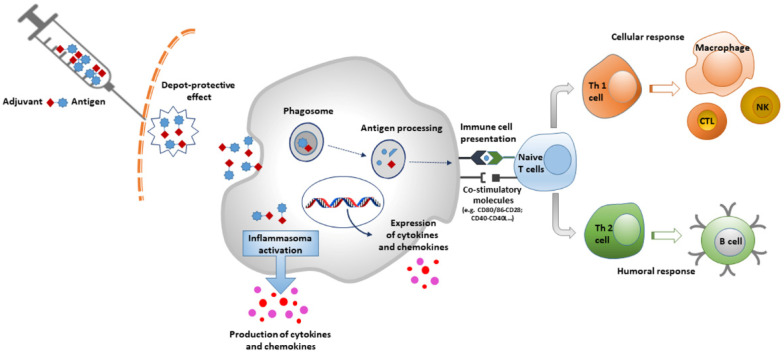
Mechanism of action of Adjuvants.

Adjuvants can be grouped according to different criteria, such as their physicochemical properties, origins, and mechanisms of action [6]. One of the most followed classification systems is the one based on their mechanisms of action, dividing them into two main categories: delivery systems (particulate) and immune potentiators [7]. A further class of adjuvants is mucosal adjuvants, a group of compounds that shares some features with the previous ones. In delivery system adjuvants, antigens are associated with an adjuvant that works especially as an antigen carrier. In addition, they are able to induce a local proinflammatory response by activating the innate immune system, leading to the recruitment of immune cells to the site of injection [8]. Specifically, the antigen-adjuvant complex activates pattern-recognition receptor (PRR) pathways by acting as pathogen-associated molecular patterns (PAMPs). This causes the activation of innate immune cells with the production of cytokines and chemokines. The same pathway is directly activated by immune potentiators [9] (Figure 1).

The addition of adjuvants is particularly useful for vaccines used in the elderly due to the physiological phenomenon of immunosenescence occurring in this category of subjects, which is responsible for the reduction of immune responses after natural infections or artificial stimuli (vaccinations) [10]. In this case, the presence of adjuvants can represent a valid tool to overcome this limit in the use of vaccines. Moreover, adjuvants are particularly useful for subunit vaccines that are often too weak to stimulate a robust immune response alone [11]. However, not all vaccines need adjuvants. For example, licensed conjugated meningococcal vaccines do not contain adjuvants because the conjugation itself with a protein carrier is able to stimulate a good immune response [12]. The currently licensed adjuvanted vaccines are listed in Table 2.

As shown by the table, the vast majority of vaccines currently licensed by EMA and FDA for human use include aluminum salts as an adjuvant. This is important to highlight, given that these adjuvants are the oldest used in vaccine formulations and the need to increase the number of new adjuvants appears absolutely necessary in order to improve vaccine safety and efficacy. For these reasons, it is essential to strengthen the research of new molecules and factors with adjuvant properties and to increase the number of in vitro and in vivo studies. At the same time, the approval of new products can suffer delays and high costs due to regulatory challenges regarding the use and the study of adjuvants, the use of new cellular substrates, or the application for process changes or transfers. This aspect can represent a barrier that stifles novelties, increases costs, and delays the availability of vaccines, especially in low-resource countries.

Moreover, the COVID-19 pandemic highlighted the importance of having access to effective vaccines in order to front the potential threat of a new pandemic. In fact, licensed RNA vaccines against COVID-19 have intrinsic adjuvant properties linked to the liposomal components used as carriers of codifying RNA. However, the newest licensed COVID-19 vaccine is based on a classical platform containing the spike protein and is adjuvanted with the addition of a new adjuvant named Matrix-M that contains fraction-A and fraction-C of *Quillaja saponaria* Molina extract [13].

Many aspects should be considered when choosing a vaccine adjuvant, among which safety is undoubtedly the first one. A good adjuvant must be principally safe, well-tolerated, and easy to produce; have good pharmaceutical features (pH, osmolality, endotoxin levels, etc.) and durable shelf life over time; and finally, be economically viable [14]. Respecting all these features without also affecting the safety of the adjuvant is difficult. For this reason, very few vaccine adjuvants are included in currently used vaccines.

Despite the great achievements obtained with vaccines, many concerns have arisen in recent decades about these products. A culture against vaccines, known as “vaccine hesitancy”, has spread worldwide. This has been encouraged by the COVID-19 pandemic, which, on one hand, has brought out the importance of vaccinations as an essential weapon against infectious diseases, but, on the other hand, has highlighted the hesitant behaviours of a certain part of the population [15,16,17]. Several causes for this attitude have been reported, but many studies have shown that the fear of side effects and mistrust regarding vaccine compositions are the most reported ones [18,19,20]. In particular, adjuvants are components that, more than others, have always aroused concerns in the general public. In actuality, when present, the side effects of vaccines are generally mild and transient and commonly represented by local pain and erythema at the site of injection, mild general malaise, and flu-like symptoms. These effects go normally away a few hours or days after vaccine administration. Very few cases of anaphylaxis or other severe side effects have been reported [21,22].

The aim of this review is to examine the currently used vaccine adjuvants and to evaluate the ongoing studies about the properties and possible future use of new adjuvants, highlighting the evidence regarding potential concerns and side effects present in the scientific literature.

## 2. Delivery Systems

### 2.1. Mineral Salts

#### Aluminium Salts

The adjuvant properties of aluminium salts were discovered in the 1920s, and these compounds have been used as vaccine adjuvants since 1926. The use of aluminium salts added to growth media was originally considered in order to induce the precipitation of tetanus and diphtheria antigens and therefore to help their purification. However, it was immediately evident that these aluminium-precipitated antigens showed more immunogenicity than the soluble ones [23]. Therefore, aluminium salts are the adjuvants that have been used for the longest period of time and the most frequently included in vaccines, with about one-third of currently licensed vaccines containing aluminium [24]. As a result, aluminium salts are the most tested in terms of safety among the vaccine adjuvants.

Humans are exposed to aluminium through different sources, especially food and air. It is absorbed into the human body mostly through the digestive and respiratory tracts, with subsequent diffusion and then a three-step elimination process, although this is never complete. Less than 3% of inhaled aluminium and 1% of ingested aluminium diffuse throughout the organism. However, ingestion through contaminated food is responsible for 95% of all aluminium that can be found in humans. [25,26]. The World Health Organization (WHO) established that the maximum level of aluminium ingested through food intake should be 1 mg/kg/day (60 to 70 mg/day for adults) [27]. Finally, aluminium can be contained in parenteral solutions and therefore can be injected, spreading throughout the body through the blood and the various body fluid compartments. In this case, in order to avoid aluminium accumulation, its level in parenteral solutions should be <25 g/L [28].

Once absorbed into an organism, aluminium spreads within body tissues. Most of the metal is then stocked in the bones, liver, lungs, and nervous system. In people with chronic kidney disease, aluminium cannot be eliminated and accumulates over time, especially in the bones and nervous system. In this category of patients, high levels of aluminium can be deposited in the brain, resulting in encephalopathy. Aluminium penetration into the brain has been accurately quantified in an in vivo experiment following the injection of the isotope aluminum-26 (^26^Al) in rats [29]. Under physiological conditions, the brain penetration of aluminium was quantified from 0.001% to 0.005% per gram of brain tissue, independent from its route of administration and chemical form. In 2010, Goullé et al. [30] quantified aluminium levels in human tissues using a technique combining inductively coupled plasma with detection by mass spectrometry in 20 deceased patients who were not previously exposed to the metal and who had not received any treatment containing aluminium or other trace minerals. The median results expressed as wet weight were as follows: lungs = 0.47 g/g, brain = 0.19 g/g, liver = 0.15 g/g, heart = 0.10 g/g, muscle = 0.08 g/g and kidney = 0.06 g/g.

Aluminium is slowly excreted by the organism, mainly through the urinary system. Some aluminium is permanently deposited in the human body, with an amount that increases with the exposure level and age. The amount of this permanently deposited aluminium has been assessed at 30 to 50 mg in adults [26].

In vaccines, aluminium is present as complex polymers of crystalline aluminium oxyhydroxide (AlH) or amorphous aluminium hydroxyphosphate (AlP) forming clustered nanoparticles. AlH has the appearance of needle-like nanoparticles (Ø 20 nm) [31], while AlP appears as a mesh when observed under a transmission electron microscope [32]. Both forms are generally solubilised in citrate, but AlP is more soluble than AlH. Antigens are adsorbed onto the surface of adjuvant particles through electrostatic interactions and ligand exchange [33]. Aluminium salt/antigen binding enhances antigen uptake and presentation by antigen-presenting cells (APCs) [34]. Furthermore, aluminium salts stimulate the activation of the NLRP3 inflammasome, resulting in the production of IL-1β and IL-18, with consequent local inflammation and recruitment of APCs [35,36].

Many vaccines use antigens adsorbed on AlH or AlP (e.g., vaccines against diphtheria and tetanus, acellular pertussis, hepatitis B, and pneumococcal and meningococcal vaccines) because of their poor immunogenic power and the subsequent need to potentiate the immune response in order to elicit an effective vaccination. In Europe, the amount of aluminium in vaccines is set by the European Pharmacopoeia to a maximum of 1.25 mg aluminium per dose [37]. In the US, the US Code of Federal Regulations set the quantity of aluminium in biological products (including vaccines) to 0.85 mg/dose [38]. In contrast to the aluminium present in food mainly in form of soluble citrate or chloride salts, the inorganic aluminium compounds used as adjuvants are poorly soluble; this is part of their mode of adjuvant action. Therefore, due to this poor solubility at physiological pH, the absorption rate of aluminium contained in vaccines after intramuscular or subcutaneous injection is expected to be very slow.

Some in vivo studies have been conducted in order to evaluate the kinetics of aluminium after intramuscular injection. An experimental study performed by Flarend et al. [39] based on intramuscular injection of AlH and AlP labelled with ^26^Al with a total dose of aluminium of 0.85 mg showed absorption rates of 17% for AlH and 51% for AlP within 28 days of experiments. The increased maximum serum concentration (Cmax) of ^26^Al was 2 μg/L, i.e., 7% of the normal value (30 μg/L) found in rabbits. On the basis of these results, it is possible to evaluate the expected increase of aluminium Cmax following intramuscular injection of aluminium salt vaccine adjuvants that would be equal to 0.04 μg/L, i.e., 0.8% of the mean blood aluminium level of 5 μg/L. In this experiment, the aluminium levels in the brain were also evaluated with concentrations between 10^−8^ and 10^−7^ mg/g, i.e., between 10^−5^ and 10^−4^ μg/g, and thus <0.0001 μg/g; this is more than 2000 times lower than the mean concentration of 0.2 μg/g present in the human brain. Furthermore, some studies have evaluated aluminium excretion in humans after the injection of ^26^Al-citrate. A rather old study showed that, after intravenous injection of ^26^Al-citrate, 59% of the injected dose was excreted in urine within one day, with slower excretion in the following days (mean retention rate of 27% on day 5) [40], while a more recent study showed that eight years after an injected dose of aluminium, the percentage of retention was about 2% [41].

The toxicity of aluminium is secondary to an increase in metal levels in body fluids and tissues. This increase is especially due to an altered capacity to eliminate it, especially when renal function is compromised. Patients with renal failure on haemodialysis are especially at higher risk of having higher aluminium levels with possible neurotoxicity. Renal transplantation is able to solve the aluminium surplus and, possibly, the associated neurotoxicity [42,43]. For many years, scientists have been debating the possible role of aluminium neurotoxicity as a cause of neurodegenerative disease even if, to date, no certain evidence has been shown and this role remains controversial.

The neurotoxicity of aluminium has been studied in vitro, ex vivo, and in vivo in animal models and humans. Some in vitro studies were conducted on bacteria, showing an absence of mutagenicity [44,45]. Furthermore, having used strains sensitive to oxidizing mutagens, doubt arises regarding the oxidative mode of action for aluminium. Other in vitro studies have been conducted on cell lines to evaluate the possible genotoxicity of different forms of aluminium [46,47,48]. Some scientists have dedicated themselves to ex vivo studies, using lymphocytes from multiple donors [49,50], embryotoxic studies in animal models [51], and finally, various studies conducted in vivo [52,53]. In all these studies, the most commonly used techniques were the comet assay and the micronucleus assay. It is interesting to note that the results are often inconsistent and contradictory, and there may be methodological flaws. For this reason, to date, it is not possible to state with certainty that aluminium salts used as adjuvants (at the recommended doses) can have toxic effects, despite their ability to create more or less intense oxidative stress [54].

### 2.2. Emulsions

The progenitors of this important group of adjuvants are complete and incomplete Freund’s adjuvants. Both these adjuvants are water-in-oil emulsions able to carry antigens and stimulate the innate immune system. Complete Freund’s adjuvant (CFA) includes in its structure heat-killed mycobacteria, which enhances the stimulation of immune responses and is currently used in in vivo experiments in order to induce strong immune activation and autoimmunity (such as uveitis and experimental autoimmune encephalomyelitis) in mice. However, CFA is able to induce a strong, long-lasting local inflammation that may result in remarkable pain to the animal, with the possible onset of an ulcer at the site of injection [55]. Incomplete Freund’s adjuvant (IFA), which does not contain mycobacteria, was used in the 1950s as an adjuvant in human influenza vaccines; it can induce stronger, long-lasting antibody responses when compared to the same vaccine without the adjuvant [56]. The adjuvant activity of IFA is based on its feature as an oily antigen deposit from which there is a continuous release of the antigen at the injection site. This leads to at the same time an increase in antigen lifetime, and strong local innate immune stimulation with phagocytosis, leukocyte recruitment and infiltration, and cytokine production [57]. However, the introduction of IFA in vaccine formulations and its regular use in humans is hindered by the elicited strong side effects. In particular, toxicity is caused by the high levels of non-biodegradable used oils as well as their poor quality [58]. A 2005 survey conducted by the WHO showed that the immunisation of about one million subjects with IFA was burdened by the onset of severe side effects, such as sterile abscesses, in 40,000 immunised people [59].

#### 2.2.1. MF59

MF59 is a water-in-oil emulsion composed of squalene, Span 85, and Tween 80 in 10 mM sodium citrate buffer at pH 6.5, with an average particle size of about 165 nm [60]. It was the first oil-in-water emulsion used as an adjuvant approved for human vaccine use in Italy in 1997 [61]. It is currently included in the adjuvanted trivalent and tetravalent (TIV and QIV) flu vaccines Fluad (Seqirus), which were initially used only in people >65 years old but were later approved for in other flu risk groups such as young children and infants and, during the H1N1 pandemic vaccine, for pregnant woman and young children [60,62,63]. It has been shown that the presence of MF59 increases the effectiveness of influenza vaccines in children <2 years of age [64,65]. MF59 was also tested as an adjuvant in an HBV vaccine and was able to trigger an impressive immune response, better than that induced with aluminium [66]. Concerning the mechanism of action, MF59 has effects similar to those of aluminium salts. Depot activity at the injection site is quite negligible, as studies have shown that its half-life is 42 h [35,67]. Conversely, MF59 has the powerful ability to induce both cellular and humoral immune responses, including the production of high titres of functional antibodies [68]. The presence of MF59 stimulates local innate immune cells to secrete chemokines such as C-C Motif Chemokine Ligand 4 (CCL4), C-C Motif Chemokine Ligand 25 (CCL2), C-C Motif Chemokine Ligand 5 (CCL5), and C-X-C Motif Ligand 8 (CXCL8), which in turn drive leukocyte recruitment, antigen uptake, and migration to lymph nodes with the triggering of the adaptive immune response [69,70]. In addition, studies have reported that MF59 is able to increase the expression of the gene cluster regulating leukocyte transendothelial migration and the subsequent recruitment of MHCII+CD11b+ cells to the injection site, eliciting a robust immune response [71]. MF59 is safe and well-tolerated, as demonstrated by millions of doses administered in over 35 countries [72].

#### 2.2.2. AS03

AS03 is an oil-in-water adjuvant emulsion composed of the surfactant polysorbate 80 and two biodegradable oils, i.e., squalene and DL-α-tocopherol in phosphate-buffered saline [73]. This adjuvant has been used for influenza vaccines, eliciting immune responses similar to MF59, as well as in malaria vaccines [74,75]. The European Commission authorized the marketing of the AS03-adjuvanted vaccine Pandemrix in 2009 [76], while an AS03-adjuvanted influenza A (H5N1) monovalent vaccine was authorized by the Food and Drug Administration (FDA) in 2013 [77]. However, the antioxidant and immunostimulatory properties of α-tocopherol would seem to enhance immune stimulation compared to MF59 [78,79]. Indeed, the use of an AS03 adjuvanted influenza vaccine in children aged from 6 to 35 months demonstrated a strong immune response, even 6 months after vaccination [80]. Some studies were performed in order to clarify the contribution of DL-α-tocopherol in AS03, comparing the effects of AS03 and a comparable emulsion lacking DL-α-tocopherol. By measuring antigen uptake, immune cell recruitment, and the levels of secreted cytokines, it was concluded that the lack of DL-α-tocopherol led to a lower immune response with lower antibody titres [79]. Moreover, it has been shown that AS03 is able to stimulate the immune system by the activation of NF-κB [5], which induces cytokine and chemokine secretion in muscle and lymph nodes and promotes the migration of innate immune cells. In addition, AS03 can stimulate CD4+ T cell-specific immune responses, which can determine long-lasting neutralizing antibody production and higher levels of memory B cells [74]. The composition of AS03 has been further supplemented with two strong immunostimulants, the QS-21 (a saponin derived from *Quillaja saponaria*) and 3-O-desacyl-4′-monophosphoryl lipid A (MPL), to boost its immunogenicity, giving rise to AS02 [81,82].

### 2.3. Microparticles

#### 2.3.1. Virus-Like Particles

Virus-like particles (VLPs) are icosahedral or rod-shaped nanoparticles (Ø 20–200 nm) consisting of a shell of self-assembling capsid protein; these have long been studied and used for vaccine development [83,84,85]. They are non-infectious particles because they do not include any genetic material. They are one of the most important representatives of a new class of vaccines, called nanovaccines, that is becoming increasingly important in vaccine development [86]. VLPs are smart nanoparticles, as they are formed by an external viral shell with repetitive epitopes that are immediately recognized as non-self by the immune system, producing strong immune responses. This feature, which it shares with natural viruses, is, however, not accompanied by the harmful capacity to cause infection. Besides these repetitive structural motifs, VLPs are similar in size to viruses (usually ranging between 20–800 nm) and undergo rapid and effective processing that leads to the production of a fast and long-lasting immune response, even in the absence of an adjuvant [87,88]. VLPs can be classified according to the presence or absence of envelopes into non enveloped VLPs and enveloped VLPs (eVLPs) [88]. Non-enveloped VLPs can be in turn divided into single or multi-capsid protein VLPs and as single-layer, double-layer, and triple-layer VLPs. A classic example of a multicapsid non-enveloped VLPs is that formed by papillomavirus L1 and L2 proteins, which are able to self-assemble to form the microparticle. eVLPs obtain their lipid membrane from the host cell in which they are expressed during assembly and budding [85] and are also sub-divided into single-layer, double-layer, and multi-layer. They can be manufactured by different viral types through different technologies using various cell systems, among which are Escherichia coli, yeasts (Saccharomyces cerevisiae and Pichia pastoris), Baculovirus, mammalian cells, plant cells, and cell-free systems [89,90]. VLP manufacturing in cell systems employs a multistep methodology called “assemble-then-purify”, with the first step exploiting the spontaneous assembling capacity of capsid proteins that occurs directly inside the expression cell vector. The next step consists of the purification of newly formed particles. Sometimes, in order to obtain well-purified particles, after cellular assembly, it is necessary to disaggregate the new particles and therefore reassemble them a second time. Another manufacturing approach uses a cell-free in vitro assembly processing system consisting of the reversal of the traditional cellular methodology [91,92,93]. In particular, an in vitro system is used as a platform to induce the spontaneous assembly of capsid proteins after their expression and purification, without the need to disassemble newly formed VLPs [94,95].

Currently, two important adjuvanted vaccines use a nanoparticle platform to induce immunisation: the hepatitis B and papillomavirus (HPV) vaccines. The currently used hepatitis B vaccine is a recombinant DNA vaccine containing hepatitis B surface antigen (HBsAg), in the form of VLPs, used to prevent hepatitis B infection and produced by recombinant DNA techniques using Saccharomyces cerevisiae as the expression vector. Each dose contains 10 µg/0.5 mL of VLPs (for children) or 20 µg/mL (for adults), both adsorbed on aluminium hydroxide hydrate [96]. The vaccine is inoculated to infants, children, and adolescents up to 15 years of age, or in groups at a high risk of acquiring hepatitis B, also showing excellent immunogenicity in neonates born from hepatitis B carrier mothers (95–99% efficacy). It appears that the HBV vaccine confers immunity for at least 10 years [97,98].

HPV vaccines are also vaccines based on the VLP platform. HPV virions are non-enveloped and contain double-stranded DNA (dsDNA). The capsid has icosahedral symmetry and is composed of major and minor structural proteins, i.e., the L1 and L2 proteins [99]. The current nonavalent HPV vaccine protects against nine different viral genotypes, which are responsible for 90% of cervical cancers and 80–95% of anogenital cancers, and its administration is recommended in both male and female subjects, starting from 9 years of age [3,100,101,102]. The nonavalent HPV vaccine contains the L1 proteins of nine different genotypes of HPV (6, 11, 16, 18, 31, 45, 53, 58) forming VLPs and synthesized by recombinant DNA technology. VLPs have the advantage of being protein structures that do not contain a viral genome and are non-infectious and non-oncogenic. The vector that is currently used for the expression of L1 proteins is Saccharomyces cerevisiae. The use of VLPs in synergy with adjuvants (AlP) allows an excellent immune response and therefore 90% protection against cervical cancer, in addition to the fact that it has been shown that the antibodies induced by the vaccine are able to cross the placenta, protecting newborns from HPV 6 and 11 [103].

#### 2.3.2. Virosomes

Virosomes are a vaccine platform very similar to the native viral structure. Structurally, they are VLPs formed by reconstituted influenza virus envelopes consisting of hemagglutinin (HA), neuraminidase (NA), and phospholipids (phosphatidylethanolamine and phosphatidylcholine) lacking viral genetic material [104]. The first use of virosomes for the manufacture of an influenza vaccine was proposed in 1975 [105]. Since then, scientific evidence on the efficacy of this kind of vaccine has become available, along with two vaccines used for the prevention of hepatitis A (Epaxal) and influenza (Inflexal) [106,107]. Inflexal V is an adjuvanted influenza vaccine suitable for all age groups and has good efficacy in both healthy and immunocompromised children, adults, and the elderly [107]. It is able to induce B cell responses and produce specific antibodies. Virosomes retain the receptor-binding capability and membrane fusion activity of viral HA but, lacking the viral RNA, they are unable to induce infection in cells after binding. Moreover, this binding capability increases their immunogenicity compared to subunit and split-virion influenza vaccines [108]. Virosomes act as a perfect delivery system, being able to move antigens into the cytosol of antigen-presenting cells (APCs) and induce cytotoxic T lymphocyte (CTL) responses [109]. However, due to their weak adjuvant properties, virosomes are not very efficient at activating APCs and promoting cross-presentation. This intrinsic limitation can be removed by adding stronger adjuvants. For example, a novel influenza vaccine based on virosomes supplemented with the Toll-like receptor 4 (TLR4) ligand monophosphoryl lipid A (MPLA) and the metal ion-chelating lipid DOGS-NTA-Ni adsorbed into the membrane was recently developed. In vitro, virosomes with adsorbed MPLA were able to induce stronger activation of APCs compared to virosomes with no added adjuvant. Moreover, in vivo immunisation of mice with these MPLA-adjuvanted virosomes resulted in the induction of specific CTLs [110].

The manufacturing of influenza virosomes includes solubilisation of the viral envelope using the detergent octa(ethylene glycol)-n-dodecylmonoether (C12E8) with subsequent ultracentrifugation and removal of viral nucleocapsids. Then, the detergent is eliminated from the supernatant with hydrophobic beads, with the subsequent reassembly of viral membrane lipids and envelope glycoproteins forming particles of approximately 100–200 nm. It has been shown that this process is able to produce influenza virosomes showing fusion properties very similar to those of the wild-type virus. Influenza virosomes enter cells through receptor-mediated endocytosis and then fuse with the endosomal membrane. Different macromolecules can be encapsulated within the virosomal lumen reaching the cytosol of target cells due to the membrane fusion activity. For instance, it was shown that DTA (the A subunit of diphtheria toxin) encapsulated within a virosome can be successfully transported into the cytosol of target cells, leading to the complete inhibition of protein synthesis [111]. Even a plasmid DNA can be encapsulated into virosomes formed by a cationic lipid. This virosome DNA can be used to efficiently transfect target cells [104].

The remarkable benefit of the virosome delivery system and adjuvants is their capacity to adsorb antigens onto their surface and lumen through hydrophobic lipid interactions. Furthermore, virosomes are preferred over VLPs in vaccine production because the latter have more limited movement due to their protein-based structure. Moreover, adsorbing antigens onto the surface of the fluid phospholipid bilayer of virosomes stimulates interactions with host cell receptors [109,112]. The FDA has approved virosomes as nanocarriers for human use due to their very high tolerance and safety profile [113,114,115]. In contrast to subunit vaccines eliciting poor responses against viral invasion, virosomes are able to induce robust humoral and cellular immunity in a very similar way to natural infection and other potent adjuvants.

To date, besides the two abovementioned virosome-based vaccines against influenza and hepatitis A, several virosome-based vaccines are under study, including those against HIV [116], HPV [117], RSV [118], and malaria [119].

HIV virosomal vaccines have shown acceptable outcomes in clinical phase Ι, and they may be available soon. Even though the vaccine can be administered by the intramuscular or subcutaneous route, the mucosal route could elicit stronger immune responses because the main route of HIV transmission is through mucosal tissue. Therefore, strong mucosal antibody production is an essential defensive mechanism against HIV infection [120]. An HIV virosome-based vaccine has been prepared from influenza viruses by adsorbing some HIV-1 virulence antigens, such as gp41 and p1 peptides, and by including the adjuvant 3M-052, a thermostable adjuvant that increases virosome membrane rigidity [121,122]. In another study, a thermostable HIV-1 virosomal vaccine was composed of an influenza-enveloped virosome with HA, NA, lecithin, cephalin, and other phospholipids, with the addition of 3M-052, Toll-like receptor (TLR7/8) and the sugar trehalose [116].

About HPV, some studies had focused on the efficacy of virosome-based vaccines containing E6 and E7 proteins fused with the host cell membrane via receptor-mediated endocytosis. Studies have shown that a recombinant HPV16 E7 influenza virosome induced strong CTL responses and prevented the development of an HPV16+ transformed cancer. In addition, immunisation with E7-virosomes induced IgG antibody responses against E7 [117].

## 3. Immune Potentiators

### 3.1. TLR1/2 Agonists

Among TLR1/2 agonists, L-pampo is a potent adjuvant system composed of Pam3Csk4 (Pam3) and polyinosinic:polycytidylic acid (polyI:C), potent TLR1/2 and TLR3 agonists respectively. In a study by Lee et al. [123], L-pampo induced a stronger antibody production against HBV than Alum and also involved cell-mediated immune responses such as increased multifunctional CD4+ T cells. Moreover, L-pampo was investigated also as a potent adjuvant against SARS-CoV-2. Specifically, SARS-CoV-2 antigens such as receptor-binding domain (RBD) and S1 antigens or RBD-Fc combined with L-pampo stimulated strong humoral and cellular immune responses against SARS-CoV-2 compared to widely used adjuvants [124].

Moreover, bacterial lipoproteins are the most potent ligands recognized by TLR2. It has been shown that synthetic lipopeptides derived from bacterial lipoproteins are strong activators of B cells and macrophages and can be used as vaccine adjuvants [125]. The 2 kDa macrophage-activating lipopetide-2 (MALP-2) from *Mycoplasma fermentans* was shown to activate immune cells through TLR2- and MyD88-dependent signaling pathways [126]. In addition to MALP-2, Pam2CSK4 and Pam3CSK4 are well recognized TLR2 agonists and they have been evaluated as therapeutic agents against infectious diseases such as leishmania [127], malaria [128], and influenza [129].

#### TLR3 Agonists

Before the discovery of TLRs, a synthetic dsRNA, polyriboisosinic:polyribocytidylic acid [poly(I:C)], was found to be highly capable of inducing IFN production [130]. TLR3, an endosomal receptor detecting viral dsRNA, recognises poly(I:C) because it structurally mimics viral RNA, thereby inducing the production of type I IFN and type III IFN, and eliciting Th1 cytokine responses [131,132,133]. Type I IFN produced after the TLR3- poly(I:C) interaction is particularly important for conventional Dendritic Cells (cDCs) to effectively activate CD8 T cell responses [134,135]. In addition, the type I IFN produced by poly(I:C) stimulates the clonal expansion of T cells, increasing the effector T cell ratio and the numbers of antigen-specific B cells [136,137,138]. For all these reasons, poly(I:C) has been widely investigated as a potential adjuvant. However, poly(I:C) has toxic effects in humans [139,140]. Hence, the attention of scientists has been focused on derivatives of poly(I:C), such as poly(ICLC) and poly(IC12U), and other synthetic TLR3 agonists such as ARNAX, IPH 3102, and RGC100. Poly(ICLC) is poly-L-lysine in carboxymethylcellulose and, similarly to poly(I:C), is able to stimulate IFN production. However, it shows higher resistance to serum nucleases, with a parallel higher immunostimulatory effect [141]. An interesting aspect of poly(ICLC) is its capacity to induce the expression of several other genetic sequences of the innate immunity pathway, including the inflammasome and the complement system AS, similar to live viral vaccines [142]. To date, some studies have used poly(ICLC) as a vaccine candidate against infectious diseases, such as *Plasmodium falciparum* [143] and HIV [144], as well as cancer [145]. It has been shown that poly(ICLC) is able to elicit a stronger Th1 immune response compared to other TLR agonists, such as LPS and CpG, which is a positive aspect in vaccination [146]. Poly(IC12U) was designed to reduce the toxicity of poly(I:C) through a mismatch between uracil and guanosine residues [147,148]. However, although this change reduced toxicity, it resulted in lower type I IFN production than poly(I:C) [149]. Unlike poly(I:C) and poly(ICLC), it has been shown that poly(IC12U) binds to TLR3 but not to MDA5 [149]. Similar to poly(ICLC), some studies have used poly(IC12U) as an adjuvant in vaccines against HIV [150], influenza [151], and cancer [152]. A new TLR3 agonist with adjuvant potential is ARNAX, a TLR3-specific ligand specifically produced to have lower toxicity than poly(I:C) [153]. The toxicity of poly(I:C) is linked to its capacity to activate the MAVS pathway (activation of RIG-I and/or MDA5) [154]. Therefore, Matsumoto et al. [154] developed a ligand including GpC phosphorothioate oligodeoxynucleotides and dsRNA, which is recognised by TLR3 and internalised into the endosome. The ligand is able to activate TLR3 while avoiding detection by MDA5 due to the relatively short length of the RNA chain. In a murine model, the adjuvant was not able to induce a significant increase in serum inflammatory cytokine levels but favoured cross-presentation of the antigen by DCs and elicited a Th1 profile [155]. The most two important fields in which ARNAX has been studied are cancer immunotherapy [156] and influenza vaccination [157].

### 3.2. TLR4 Agonists

The TLR4 agonists studied as vaccine adjuvants are AS01, AS02, and AS04, all containing the MPLA, the ligand of endosomal TLR4. Specifically, AS01 has been used to develop vaccines against malaria [158], HIV [159,160], and tuberculosis [161]. AS01 is a combined adjuvant system consisting of two different immunostimulatory molecules, MPLA and QS-21, encapsulated in a liposome structure [82]. QS-21 is a natural triterpene glycoside saponin extracted from the bark of *Quillaja saponaria Molina* [162]. These two compounds use liposomes as a carrier to reach cells through cholesterol-dependent endocytosis [163]. Inside the cell, QS-21 causes lysosomal destabilisation and promotes the activation of the protein kinase SYK [163]. MPLA links endosomal TLR4, inducing the TRIF-dependent signalling pathway [164]. QS-21 used alone has an important and adverse haemolytic effect, inducing cell death [165]. However, the haemolytic activity of QS-21 and the consequent cell death is abrogated by encapsulation in liposomes [166]. AS01 activates caspase-1 and thereby promotes NLRP3 inflammasome activation and the release of IL-1β as well as IL-18 from APCs [167]. The release of IL-18 causes the rapid production of IFN-γ, especially by natural killer cells in the lymph nodes, thereby promoting the maturation of DCs and the induction of a Th1-type immune response [168].

### 3.3. TLR5 Agonists

TLR5 is a receptor recognising bacterial flagellin and is expressed by several immune cells. The link with the ligand causes the activation of inflammation pathways and the release of many inflammatory mediators such as TNF-α, IL-1β, IL-6, and nitric oxide [169]. Moreover, flagellin is able to evoke both Th1 and Th2 responses, unlike other TLR ligands that are only capable of inducing, above all, Th1 responses [170]. In addition, flagellin induces the production and release of IL-1β through the activation of the NLRC4 inflammasome [171,172]. Flagellin is able to exert adjuvant activity in a TLR5- or NLRC4-independent model, but with lower efficiency than the wild type. Indeed, the adjuvant capacity is greatly decreased when both the receptors are not present in a murine model, which suggests that at least one of the receptors needs to be present in order to drive an immune response; the presence of both provides the best immunisation results [173,174]. It has been shown that flagellin maintains its adjuvant capacity in immunocompromised people, for example in HIV+ patients [175]. Cui et al. [176] reviewed all the studies using flagellin as an adjuvant. The simplest method is administering it with an antigen; this simple method successfully induces a mucosal immune response essential in protecting against respiratory and gastrointestinal infections [177,178]. Many studies have been carried out, especially regarding the role of flagellin as an adjuvant in influenza vaccines [179,180,181,182,183]. In these studies, flagellin from *Salmonella typhimurium* was used combined with different influenza antigens, among which inactivated PR8 influenza virus (IPR8), HA(H5N1), and avian influenza virus (AIV) H5N1, and for each of them, a robust immune response (especially mucosal with IgA production) was obtained. Flagellin can also be successfully modified in order to obtain chimeric flagellins or complexes of flagellin antigen in live attenuated bacteria such as *Mycobacterium tuberculosis* [178], *Vibrio cholerae* [184], *Streptococcus pyogenes* [185], *Listeria monocytogenes* [186] and enterotoxigenic *Escherichia coli* (ETEC) [187,188]. Moreover, the production of recombinant flagellin-antigen fusion proteins has been used in animal models as adjuvanted vaccines both for infectious diseases and cancers [176]. To date, at least three vaccines using flagellin as the adjuvant are in the clinical trial phase: two against the influenza virus and one against *Yersinia pestis* [181,189,190].

### 3.4. TLR7/8 Agonists

Some studies have shown that agonists of TLR7/8 are able to strongly induce a Th1 immune response [191,192]. Ligand binding to TLR7/8 produces high levels of type I IFN, IL-12, TNF-α, and IL-1β. In addition, TLR7/8 and TLR9 agonists are the only agonist molecules capable of activating and promoting the clonal expansion of both cDCs and Plasmacytoid dendritic cells (pDCs), also mobilising CD14+CD16+ inflammatory monocytes and CD14dimCD16+ patrolling monocytes [192]. The most important representative TLR7/8 agonists are some synthetic small molecules named imiquimod (R837) and resiquimod (R848), which belong to the class of imidazoquinolines [193]. Imiquimod is currently approved and licensed for the treatment of genital warts, superficial basal cell carcinoma, and actinic keratosis, while resiquimod has been studied for its antiviral and anticancer therapeutic use. However, these small molecules have been shown to have some intrinsic limits. In particular, they can spread away from the site of administration and thus far from the antigen, thereby decreasing efficacy and inducing systemic side effects [194]. Therefore, it has been shown that a direct conjugation of these molecules to aluminum adjuvants is able to improve vaccine efficacy [195]. Some previous studies carried out the direct conjugation of imidazoquinolines to HIV-1 Gag protein or whole inactivated influenza viruses, increasing Th1 responses and the number of antigen-specific T cells [196,197,198]. Moreover, conjugation to synthetic polymer scaffolds, lipid-polymer amphiphiles, polyethylene glycol (PEG), nanogels, alum, and various other synthetic polymers remarkably increased the delivery of imidazoquinolines and improved the maturation of DCs and antigen-specific T cells [199]. Moreover, previous studies using a mix of imidazoquinolines with one or more other TLR agonists, such as MPLA (TLR4) and MPLA + CpG ODN (TLR4 and TLR9), showed that this combination increased innate immune responses, with remarkable production of antigen-specific neutralising antibodies and improved Th1 responses [200,201,202]. All of these innovative aspects highlight the excellent potential of TLR7/8 agonists as adjuvant candidates.

### 3.5. TLR9 Agonists

TLR9 naturally recognises the bacterial DNA motifs represented by the unmethylated cytosine-phosphate-guanine (CpG) dinucleotide, driving the activation of the innate immune system through the MyD88-dependent pathway [203]. These molecular motifs have been used as synthetic adjuvants with specific modifications in order to prevent degradation by nucleases [204]. CpG-ODNs cause robust chemokine, cytokine and antibody production in natural killer cells, B cells and pDCs, driving a vigorous Th1-type immune response [205]. To date, three different classes of CpG-ODN ligands belonging to three classes (A-C) have been developed, but only molecules belonging to class B have been used in a clinical trial as adjuvants [206]. CpG-B ODN localises to endosomes and causes the maturation of pDCs [207]. Moreover, CpG-B ODN can directly interact with B cells to enhance antibody production [208]. It has been shown in a murine model that CpG-B ODN as an adjuvant causes considerable and long-lasting antibody production, better than alum-adjuvated or non-adjuvated vaccines [209]. The recently licensed CpG 1018, an oligonucleotide with high chemical stability and adjuvant capacity to elicit Th1-type immune responses, is used as adjuvant in the hepatitis B vaccine Heplisav-B [210]. CpG 1018 in Heplisav-B improves vaccine efficacy, requiring a schedule consisting of only two doses compared to conventional hepatitis B vaccines needing three doses to elicit the best protection [210]. To date, CpG 1018 is under study for the development of several vaccines, including those against melanoma [211] and COVID-19 [212]. Another CpG ODN, CpG 7909, is also under clinical evaluation and has shown encouraging results in HBV and malaria vaccinations [213]. Other next generation TLR9 agonists have been developed. A valid representative is MGN1703, a small DNA molecule that includes CG motifs but is structurally different from CPG ODN. MGN1703 is formed by a section of reverse complementary DNA that is double-stranded in the middle and bordered by two single-stranded loops that include three non-methylated CG motifs, forming a dumbbell-shaped structure in contrast to CpG ODNs, which are linear molecules [214]. MGN1703 has been tested as an adjuvant in vaccines against cancers and it has been found that it is able to activate both innate and adaptive immune responses with only mild or temporary side effects [214,215].

## 4. Potential Side Effects of Adjuvants: The Asia Syndrome

Despite the excellent safety of vaccines, in recent years, a new concern has arisen about their possible negative effects and, next to the well-known abovementioned side effects, a new nosological entity was described. The entity is autoimmune/inflammatory syndrome induced by adjuvants (ASIA), which was first presented by Shoenfeld et al. [216]. This syndrome includes some immune-mediated disorders, which are likely to occur in genetically susceptible individuals after their exposure to adjuvants. The features of this syndrome are mainly the production of autoantibodies and improvement once the triggering agent is removed [217]. At the onset of this syndrome, some external factors such as infectious agents or adjuvants (i.e., dust, silicone, aluminium salts, etc.) act on a predisposing genetic background mediated by particular HLA antigens associated with the development of autoimmune diseases (ADI) [218,219,220,221]. In particular, the simultaneous presence of the HLA-DRB1 and the PTPN22 gene has been shown to be the most common autoimmune background in these patients [222,223]. According to recent scientific evidence, some pathologic conditions such as sarcoidosis, Sjögren syndrome (SS), undifferentiated connective tissue disease (UCTD), silicone implant incompatibility syndrome (SIIS), and immune-related adverse events (irAEs) are typical examples of the ASIA syndrome context [224]. In addition to the common adjuvants contained in vaccines, many other substances such as silicone, paraffin, hyaluronic acid, acrylamides, and methacrylate show adjuvant properties [225,226].

According to Watad et al. [217], there are two types of criteria that can aid in the diagnosis of ASIA, distinguished into major and minor criteria. The major criteria include exposure to various exogenous stimuli (infection, contact with adjuvants) prior to clinical manifestations and the appearance of typical clinical manifestations like myalgia, myositis, arthralgia, arthritis, chronic fatigue, sleep disturbances, demyelination, memory loss, pyrexia, and dry mouth. Minor criteria include the appearance of autoantibodies or antibodies directed against the adjuvant, the presence of specific HLA patterns (i.e., HLA DRB1, HLA DQB1), and the evolution of an autoimmune disease, i.e., multiple sclerosis or systemic sclerosis.

Some previous studies have reported that vaccines containing aluminium salts have the ability to cause the onset of ASIA [227]. An example is represented by the quadrivalent vaccine for HPV (containing aluminium salts), which has been reported to increase the risk of self-immunity in susceptible subjects a few weeks after vaccination [228], or the HBV vaccine [229]. However, an important study by Linneberg [230] about the potential side effects of aluminium salts showed that people undergoing subcutaneous allergy immunotherapy with multiple administrations of allergens combined with aluminum hydroxide as adjuvant and then receiving an amount of aluminum about 100 times higher than that included in a three-dose vaccine, had lower mortality and developed fewer autoimmune diseases than a control group that received conventional allergy therapy.

Scientists’ efforts have turned towards research into biomarkers to diagnose ASIA or to predict a predisposition to it (in addition to what has already been stated previously). For example, ACE 1 and IL-2 receptors increase by 50% in subjects suffering from this syndrome, and a deficiency in vitamin D increases the incidence of ASIA (due to the lack of immunomodulatory effect) [231]. It is important to emphasise how predisposition seems to be very relevant in the onset of ASIA, which was well-highlighted by Watad et al. [232] through the analysis of 500 cases of ASIA syndrome. This study showed how a higher rate of female individuals, smokers, and those with previous autoimmune diseases or with family members affected by the latter fall ill with ASIA. Polygenic autoimmune diseases were the most common among these, and UCTD and Sjögren’s syndrome had the highest prevalence at 38.8% and 16.8%. Out of the 54.4% of patients with a positive autoantibody test, 48.2% were ANA positive. It is obvious, considering that behind the development of an autoimmune/autoinflammatory state, there are environmental and genetic factors. The median time between exposure to vaccination and the onset of symptoms was one week (2 days to 5 years); 48.2% of the population developed clinical symptoms after exposure to at least one vaccine. However, next to this scientific evidence, some studies showed no relationships between the administration of adjuvanted vaccines and the ASIA syndrome [233,234]. In these studies, it has been shown that the association between vaccination and autoimmunity is probably spurious for the presence of confounding factors and the result of random events rather than a real causal relationship. The possible link between vaccine administration or exposure to foreign material and the potential occurrence of autoimmune/inflammatory and immune-mediated events should not be a ‘false myth’ that reduces vaccination coverage. Vaccine adverse events, indeed, very rarely occur. Due to the dearth of information and robust data, ASIA is an adequate umbrella term to gather together events and apparently unrelated reactions, which share exposure to vaccines, silicone, or other foreign material as the common root [217,235]. It is important to underline that, even if future research shows a real correlation between adjuvants and autoimmunity, this would nevertheless not diminish the huge and undoubted protective role played by vaccine immunisation practices, which offer many clinical benefits; in fact, vaccines have contributed to the eradication and control of numerous communicable diseases, improving the quality of human life.

## 5. Conclusions

Vaccinations have been and continue to be one of the most powerful weapons in the hands of humanity in the fight against infectious diseases. Thanks to these effective and safe preventive tools, humans have been able to eradicate the most terrible enemies in the history of humankind from many parts of the world. The importance of vaccinations has been well-highlighted by the recent COVID-19 pandemic, and further progress must be made by vaccine research in preparation for future pandemics. The efficacy of vaccines is based on the essential properties and contribution of adjuvants. In regard to the future of vaccinations, more attention must be given to these molecules in order to produce increasingly safe and effective vaccines. Research in this field is ongoing, and several products are under study to reach this goal for the benefit of humanity.

## Figures and Tables

**Table 1:** Classification of adjuvants according to their main mechanism of action.

Adjuvant Groups	Types of Adjuvants
***Delivery systems***	
*Mineral Salts*	Aluminium salts
*Emulsions*	Freund’s adjuvants
	MF59
	AS03
*Microparticles*	Virus-like particles
	Virosomes
	PLA/PLGA
***Immune Potentiators***	
*TLR1/2 agonists*	L-pampo, MALP-2, Pam2CSK4 and Pam3CSK4
*TLR3 agonists*	Poly(I:C) (polyinosinic:polycytidylic acid)
	Poly-ICLC
*TLR4 agonists*	Monophosphoryl lipid A (MPL)
*TLR5 agonists*	Flagellin
*TLR7/8 agonists*	Imiquimod (R837; 1-(2-methylpropyl)-1H-imidazo [4,5-c]quinolin-4-amine) and resiquimod (R848, 4-amino-2-(etoximetil)-a,a-dimethyl-1H-imidazo [4,5-c]quinoline-1-ethanol)
*TLR9 agonists*	CpG ODNs
***Combined adjuvants***	AS01 and AS02
	AS04
***Mucosal adjuvants***	Cholera toxin (CT)
	Heat-labile enterotoxin (LTK3 and LTR72)
	Chitosan

**Table 2:** Adjuvanted vaccines currently licensed by FDA and EMA for human use.

Trade Name	Type	Adjuvant	Administration
***ADACEL***	Tetanus Toxoid, Reduced Diphtheria Toxoid and Acellular Pertussis Vaccine	Aluminum phosphate (0.3 mg Al^3+^)	Intramuscular
***AVAXIM***	Inactivated hepatitis A vaccine	Aluminium hydroxide (0.3 mg Al^3+^)	Intramuscular
***BEXSERO***	Group B meningococcal vaccine (recombinant)	Aluminium hydroxide (0.5 mg Al^3+^)	Intramuscular
***BIOTHRAX***	Anthrax Vaccine Adsorbed	Aluminium hydroxide (0.6 mg Al^3+^)	Intramuscular/ Subcutaneous
***BOOSTRIX***	Diphtheria, tetanus and pertussis (acellular component) vaccine (reduced antigen content)	Aluminium hydroxide, (0.3 mg Al^3+^) and Aluminum phosphate (0.2 mg Al^3+^)	Intramuscular
***CERVARIX***	Human Papillomavirus vaccine (types 16, 18) (recombinant)	AS04 containing 3-*O*-desacyl-4’-monophosphoryl lipid A (MPL) 50 μg adsorbed on Aluminum hydroxide (0.5 mg Al^3+^)	Intramuscular
***DAPTACEL***	Diphtheria & Tetanus Toxoids & Acellular Pertussis Vaccine Adsorbed	Aluminum phosphate (0.3 mg Al^3+^)	Intramuscular
***DIFTETALL***	Diphtheria and tetanus vaccine (reduced antigenic content)	Aluminium hydroxide (1.5 mg)	Intramuscular
***ENGERIX B***	Hepatitis B (recombinant) vaccine	Aluminium hydroxide, (0.25 mg Al^3+^)	Intramuscular
***FENDRIX***	Hepatitis B (recombinant) vaccine	AS04 containing 3-*O*-desacyl-4′-monophosphoryl lipid A (MPL) 50 μg adsorbed on Aluminum hydroxide (0.5 mg Al^3+^)	Intramuscular
***FLUAD***	Inactivated influenza vaccine, surface antigen	MF59 containing 9.75 mg of squalene; 1.175 mg of polysorbate 80; 1.175 mg of sorbitan trioleate; 0.66 mg of sodium citrate; 0.04 mg of citric acid.	Intramuscular
***GARDASIL***	Human Papillomavirus Quadrivalent (Types 6, 11, 16, 18) Vaccine (recombinant)	Amorphous aluminum hydroxyphosphate sulfate (0.225 mg Al^3+^)	Intramuscular
***GARDASIL 9***	Human Papillomavirus 9-valent vaccine (recombinant)	Amorphous aluminum hydroxyphosphate sulfate (0.5 mg di Al^3+^).	Intramuscular
***HAVRIX***	Inactivated hepatitis A vaccine	Aluminium hydroxide (0.5 mg Al^3+^)	Intramuscular
***HBVAXPRO***	Hepatitis B vaccine, purified antigen (recombinant)	Amorphous aluminium hydroxyphosphate sulfate (0.25 mg Al^3+^)	intramuscular
***HEPLISAV-B***	Hepatitis B Vaccine (recombinant)	CpG 1018	Intramuscular
***HEXYON***	Diphtheria, tetanus, pertussis (acellular component), hepatitis B (recombinant, poliomyelitis (inactivated) and *Haemophilus influenzae* type b conjugated vaccine	Aluminium hydroxide (0.6 mg Al^3+^)	Intramuscular
***IMOVAX*** ***TETANO***	Tetanus vaccine	Aluminium hydroxide (0.6 mg Al^3+^)	Intramuscular
***INFANRIX HEXA***	Diphtheria, tetanus, pertussis (acellular component), antihepatitis B (recombinant), polio (inactivated) and anti-*Haemophilus influenzae* type b conjugate vaccine.	Aluminium hydroxide (0.5 mg Al^3+^)	Intramuscular
***IXIARO***	Japanese Encephalitis Virus Vaccine, Inactivated, Adsorbed	Aluminum hydroxide (0.25 mg Al^3+^)	Intramuscular
***KINRIX***	Diphtheria and Tetanus Toxoids and Acellular Pertussis Adsorbed and Inactivated Poliovirus Vaccine	Aluminum hydroxide (0.6 mg Al^3+^)	Intramuscular
***MENJUGATE***	Group C Meningococcal vaccine Conjugated	Aluminium hydroxide (0.3–0.4 mg Al^3+^)	Intramuscular
***NEISVAC-C***	Conjugated polysaccharide vaccine	Aluminium hydroxide (0.5 mg Al^3+^)	Intramuscular
***NUVAXOVID***	COVID-19 Vaccine (recombinant, adjuvanted)	Matrix-M containing Fraction-A (42.5 μg) and Fraction-C (7.5 μg) of *Quillaja saponaria* Molina extract	Intramuscular
***PEDIARIX***	Diphtheria & Tetanus Toxoids & Acellular Pertussis Vaccine Adsorbed, Hepatitis B (recombinant) and Inactivated Poliovirus Vaccine Combined	Aluminum hydroxide and Aluminum phosphate (0.85 mg Al^3+^)	Intramuscular
***PEDVAXHIB***	*Haemophilus influenzae* b Conjugate Vaccine (Meningococcal Protein Conjugate)	Amorphous aluminum hydroxyphosphate sulfate (0.225 mg Al^3+^)	Intramuscular
***PENTACEL***	Diphtheria and Tetanus Toxoids and Acellular Pertussis Adsorbed, Inactivated Poliovirus and *Haemophilus influenzae* b Conjugate (Tetanus Toxoid Conjugate) Vaccine	Aluminum phosphate (0.3 mg Al^3+^)	Intramuscular
***POLIOBOOSTRIX***	Diphtheria, tetanus, pertussis (acellular component) and poliomyelitis (inactivated) vaccine (with reduced antigen content)	Aluminium hydroxide (0.3 mg Al^3+^) and Aluminum phosphate (0.2 mg Al^3+^)	Intramuscular
***POLIOINFANRIX***	Diphtheria, tetanus, pertussis (acellular component) and poliomyelitis (inactivated) vaccine	Aluminium hydroxide, (0.5 mg Al^3+^)	Intramuscular
***PREVENAR 13***	Pneumococcal polysaccharide conjugate vaccine (13-valent)	Aluminium hydroxide (0.125 mg Al^3+^)	Intramuscular
***QUADRACEL***	Diphtheria and Tetanus Toxoids and Acellular Pertussis Adsorbed and Inactivated Poliovirus Vaccine	Aluminum phosphate (0.33 mg Al^3+^)	Intramuscular
***RECOMBIVAX HB***	Hepatitis B Vaccine (recombinant)	Amorphous aluminum hydroxyphosphate sulfate (0.25–0.5 mg Al^3+^)	Intramuscular
***REVAXIS***	Diphtheria, tetanus and poliomyelitis (inactivated) vaccine (reduced antigenic content)	Aluminium hydroxide (0.35 mg Al^3+^)	Intramuscular/ Subcutaneous
***SHINGRIX***	Herpes zoster vaccine (recombinant)	AS01B containing *Quillaja saponaria* Molina plant extract, fraction 21 (QS-21) 50 μg, 3-O-desacyl-4′-monophosphoryl lipid A (MPL) from *Salmonella minnesota* 50 μg	Intramuscular
***SYNFLORIX***	Pneumococcal polysaccharide conjugated vaccine	Aluminum phosphate (0.5 mg Al^3+^)	Intramuscular
***TDVAX***	Tetanus & Diphtheria Toxoids, Adsorbed	Aluminum phosphate (0.5 mg Al^3+^)	Intramuscular
***TENIVAC***	Tetanus & Diphtheria Toxoids Adsorbed for Adult Use	Aluminum phosphate (0.33 mg Al^3+^)	Intramuscular
***TETRAVAC***	Diphtheria, tetanus, pertussis (acellular component) and poliomyelitis (inactivated) vaccine	Aluminium hydroxide (0.3 mg Al^3+^)	Intramuscular
***TICOVAC***	Tick-borne encephalitis Vaccine (whole virus, inactivated)	Aluminium hydroxide (0.17 mg Al^3+^)	Intramuscular
***TRIAXIS***	Diphtheria, tetanus, pertussis (acellular components) vaccine (reduced antigenic content)	Aluminum phosphate (0.33 mg Al^3+^)	Intramuscular
***TRIAXIS*** ***POLIO***	Diphtheria, tetanus, pertussis (acellular components) and poliomyelitis (inactivated) vaccine (reduced antigenic content)	Aluminum phosphate (0.33 mg Al^3+^)	Intramuscular
***TRIBACCINE***	Diphtheria, tetanus and pertussis (acellular component) vaccine (reduced antigenic content)	Aluminium hydroxide (0.5 mg Al^3+^)	Intramuscular
***TRUMENBA***	Group B meningococcal vaccine (recombinant)	Aluminum phosphate (0.25 mg Al^3+^)	Intramuscular
***TWINRIX***	Hepatitis A (inactivated) and Hepatitis B (recombinant) vaccine	Aluminium hydroxide (0.05 mg Al^3+^) Aluminum phosphate (0.4 mg Al^3+^)	Intramuscular
***TWINRIX ADULTS***	Hepatitis A (inactivated) and Hepatitis B (recombinant) vaccine	Aluminium hydroxide (0.05 mg Al^3+^)	Intramuscular
***VAQTA***	Inactivated Hepatitis A vaccine	Amorphous aluminum hydroxyphosphate sulfate (0.45 mg di Al^3+^)	Intramuscular
***VAXELIS***	Diphtheria, tetanus, pertussis (acellular component), hepatitis B (recombinant), polyomyelitis (inactivated) and *Haemophilus influenzae* type b vaccine	Aluminum phosphate (0.17 mg Al^3+^) Amorphous aluminium hydroxyphosphate sulfate (0.15 mg Al^3+^)	Intramuscular

## Data Availability

Not applicable.
